# A real-world data analysis of electronic health records to investigate the associations of predominant negative symptoms with healthcare resource utilisation, costs and treatment patterns among patients with schizophrenia

**DOI:** 10.1136/bmjopen-2024-084613

**Published:** 2024-07-31

**Authors:** Rashmi Patel, Carole Dembek, Yida Won, Aditi Kadakia, Xueyan Huang, Courtney Zeni, Andrei Pikalov

**Affiliations:** 1 Department of Psychiatry, University of Cambridge, Cambridge, UK; 2 Sunovion Pharmaceuticals Inc, Marlborough, Massachusetts, USA; 3 Holmusk Technologies Inc, New York, New York, USA; 4 APILAX LLC, Kinnelon, NJ, USA

**Keywords:** schizophrenia & psychotic disorders, hospitalization, electronic health records

## Abstract

**Abstract:**

**Objectives:**

Negative symptoms in schizophrenia are associated with significant illness burden. We sought to investigate clinical outcomes for patients with schizophrenia who present with predominant negative symptoms (PNS) vs without PNS.

**Design:**

Retrospective analysis of electronic health record (EHR) data.

**Setting:**

25 US providers of mental healthcare.

**Participants:**

4444 adults with schizophrenia receiving care between 1999 and 2020.

**Exposure:**

PNS defined as ≥3 negative symptoms and ≤3 positive symptoms recorded in EHR data at the time of the first recorded schizophrenia diagnosis (index date). Symptom data were ascertained using natural language processing applied to semistructured free text records documenting the mental state examination. A matched sample (1:1) of patients without PNS was used to compare outcomes. Follow-up data were obtained up to 12 months following the index date.

**Primary outcome measure:**

Mean number of psychiatric hospital admissions.

**Secondary outcome measures:**

Mean number of outpatient visits, estimated treatment costs, Clinical Global Impression – Severity score and antipsychotic treatments (12 months before and after index date).

**Results:**

360 (8%) patients had PNS and 4084 (92%) did not have PNS. Patients with PNS were younger (36.4 vs 39.7 years, p<0.001) with a greater prevalence of psychiatric comorbidities (schizoaffective disorders: 25.0 vs 18.4%, p=0.003; major depressive disorder: 17.8 vs 9.8%, p<0.001). During follow-up, patients with PNS had fewer days with an antipsychotic prescription (mean=111.8 vs 140.9 days, p<0.001). Compared with matched patients without PNS, patients with PNS were more likely to have a psychiatric inpatient hospitalisation (76.1% vs 59.7%, p<0.001) and had greater estimated inpatient costs ($16 893 vs $13 732, p=0.04).

**Conclusions:**

Patients with PNS were younger and presented with greater illness severity and more psychiatric comorbidities compared with patients without PNS. Our findings highlight an unmet need for novel therapeutic approaches to address negative symptoms to improve clinical outcomes.

**STRENGTHS AND LIMITATIONS OF THIS STUDY**

- The use of automated natural language processing models applied to real-world data from electronic health records enabled ascertainment of predominant negative symptoms in a large sample size of patients with schizophrenia.
- The data analysed in this study included a range of clinically meaningful outcomes including psychiatric hospital admissions, outpatient visits, estimated treatment costs, illness severity and prescribed antipsychotic treatments.
- A matched study design enabled an analysis of associations of predominant negative symptoms with clinical outcomes while taking into account potentially confounding factors including demographic characteristics, illness severity, comorbid psychiatric disorders and type of antipsychotic treatment.
- Analysis of real-world data is limited by missing or erroneously recorded data and patients may have received care from other healthcare providers not represented in the analysed dataset.
- Natural language processing models do not always accurately identify documentation of symptoms in electronic health records and can only identify data that are recorded at the time of clinical assessment.

## Introduction

 Schizophrenia is a chronic psychiatric disorder with a lifetime prevalence of approximately 1% and is associated with a significant illness burden for affected individuals, their carers and society as a whole.^1 2^ Core features of schizophrenia include positive symptoms (eg, delusions and hallucinations), negative symptoms (eg, avolition, apathy and blunted or flattened affect) and cognitive dysfunction (eg, impaired working memory or verbal fluency).^3 4^ Negative symptoms may include symptoms of diminished expression or motivational symptoms and are distinct from cognitive impairment.^5 6^ Negative symptoms are experienced by at least half (50%–60%) of patients with schizophrenia^7^ and affect individuals from the initial stages of illness with first episode psychosis^8^ and early-onset psychosis^9 10^ through to chronic schizophrenia.^11^

Negative symptoms in schizophrenia are more prevalent among males and older patients^7 8^ and are highly related to social and occupational functioning.^12^ Patients with negative symptoms have significantly greater odds of unemployment.^7^ Schizophrenia is associated with high rates of comorbid psychiatric conditions such as anxiety and depressive disorders,^13 14^ which could also be associated with negative symptoms.^15^

Antipsychotic medications, the standard of care in schizophrenia, are generally effective in managing positive symptoms but have limited effectiveness in managing negative symptoms^16 17^ and may even contribute to secondary negative symptom burden.^18^ When significant negative symptoms occur in the presence of minimal or well-controlled positive symptoms, a patient may be classified as having schizophrenia with predominant negative symptoms (PNS).^16 19^

The characterisation of patients with schizophrenia with PNS and the burden of schizophrenia with PNS is not well known due, in part, to inconsistent definitions of PNS in the literature.^16 19 20^ In an earlier study based on Positive and Negative Syndrome Scale (PANSS) subscale scores, patients with ‘prominent’ negative symptoms (defined by a score ≥4 on at least three PANSS negative items [blunted affect, emotional withdrawal, poor rapport, passive-apathetic social withdrawal, difficulty in abstract thinking, lack of spontaneity and flow of conversation, stereotyped thinking] but not on positive items [delusions, conceptual disorganisation, hallucinatory behaviour, excitement, grandiosity, suspiciousness, hostility]) reported no significant difference in age, sex, race or marital status between cohorts with prominent negative, prominent positive and both prominent negative and positive symptoms.^21^ However, patients with prominent negative symptoms had worse health-related quality of life compared with patients without prominent negative or positive symptoms.^21^

The annual economic burden of schizophrenia has been estimated to be $343.2 billion in the USA, of which 18% is attributed to direct healthcare costs.^22^ The main contributor to direct healthcare costs in schizophrenia is inpatient hospitalisation.^22 23^ A small number of studies have reported an association between negative symptoms and greater healthcare resource utilisation (HCRU), including inpatient hospitalisations, as well as direct healthcare costs.^24^,^26^ However, little is known about the potential added HCRU and economic burden associated with PNS in patients with schizophrenia.

There is, therefore, an unmet need to identify patients with schizophrenia and PNS to better understand the associations of PNS with treatments and clinical outcomes. The purpose of this study was twofold. The first objective was to characterise patients with schizophrenia with PNS and estimate HCRU and costs in patients with schizophrenia with PNS compared with those without PNS. The second objective was to describe antipsychotic treatment patterns in patients with PNS compared with without PNS.

## Material and methods

### Data source

This retrospective analysis used data from the NeuroBlu platform, a database of de-identified electronic health record (EHR) data from 25 private mental healthcare centres (outpatient, inpatient, telemedicine and/or residential care facilities) in the USA that employed the MindLinc EHR system.^27^ The database covers approximately 560 000 patients treated by over 11 000 clinicians across 12 US states spanning a 21-year period (between 1999 and 2020). Around 14 500 patients in the database have received a diagnosis of schizophrenia. Further details of the mental healthcare centres included in the study are provided in a previously published cohort profile.^27^ The NeuroBlu database includes structured information such as demographics, diagnoses, medications and clinical outcome scales such as the Clinical Global Impressions – Severity of Illness scale (CGI-S)^28^ as well as semistructured information such as mental state examination (MSE) and treatment plans. The MSE contains the clinician’s assessment of clinical features of mental disorders which may be observed by the clinician or elicited through direct questioning or cognitive tasks. After completing the MSE during a clinical assessment, clinicians document their findings as semistructured free text in the MindLinc EHR system.

### NLP-derived symptom data

Natural language processing (NLP) algorithms have been shown to be useful to obtain data on symptoms when semistructured and/or unstructured text is used to record a patient’s mental and behavioural health state.^29^ NLP is particularly useful to obtain data that may not be recorded in a standardised way within an EHR dataset. For example, in routine clinical practice, clinicians may seldom employ structured rating scales to assess negative symptoms at every clinical assessment but may document the presence of problematic negative symptoms in free text in the MSE section of the EHR. The use of NLP therefore enables the ascertainment of negative symptoms in real-world EHR data where they are documented in free text but where no negative symptom rating scale has been applied. Numerous studies have been published using NLP to ascertain symptoms from EHR data.^89 11 29^,^33^

We applied previously developed NLP models to identify the presence of seven positive (hallucinations including visual or auditory; delusions including paranoia, grandeur, obsession or unspecified; responding to internal stimuli) and seven negative symptoms (blunted affect, appearance, attention and concentration, cognition, language, psychomotor, speech) recorded within semistructured MSE data in the NeuroBlu database using a deep learning approach.^34^ In brief, MSE data on 241 clinical features were reorganised into 27 categories representing key features of mental disorders (eg, ‘Abnormal or psychotic thoughts’, ‘Mood’, ‘Speech’ etc). Free text data recorded in the MSE were tokenised and pre-processed to remove common parts of speech and correct spelling errors. A training dataset was analysed using a long short-term memory approach with fivefold cross-validation. Models for NLP-derived clinical features were validated against pre-labelled test data. The median AUROC across all clinical features was 0.9 indicating good classification accuracy.

Of the 241 clinical features available for analysis, 22 features across 10 categories that were relevant to positive and negative symptoms were ascertained for this study. A summary of the symptom data obtained in the study is included in online supplemental etable 1. In addition to negative symptoms related to avolition and diminished emotional expression, we chose to include the domain of ‘Attention and concentration’ on the basis of its inclusion in the Scale for the Assessment of Negative Symptoms.^35^

### Ethical considerations

The NeuroBlu database is de-identified and standardised to a common data model which conforms with Observational Health Data Sciences and Informatics data standards. The NeuroBlu database has received a waiver of authorisation for analysis of de-identified healthcare data from the WCG Institutional Review Board (Ref: WCG-IRB 1-1470336-1).

### Patient and public involvement

Patients and the public were not involved in the design, or conduct, or reporting, or dissemination plans of the research study.

### Study participants

Adult patients (age ≥18 years) were eligible for inclusion in the study if they had a diagnosis of schizophrenia (ICD-9=295.00–5, 295.10–5, 295.20–5, 295.30–5, 295.40–5, 295.50–5, 295.60–5, 295.80–5, 295.90–5; ICD-10=F20.0–3, F20.5, F20.81, F20.89, F20.9), were treated at a mental healthcare centre with inpatient facilities and received antipsychotic treatment. The earliest date of schizophrenia diagnosis in the health records was defined as the index date. Patients were excluded if they did not have at least 1 year of health records after the index date or a CGI-S measurement within 14 days before or after the index date. Patients were then categorised as having schizophrenia with PNS if they had ≥3 recorded negative symptoms and ≤3 recorded positive symptoms at index (PNS cohort) or schizophrenia without PNS if they had <3 recorded negative symptoms or >3 recorded positive symptoms at index (non-PNS cohort). The thresholds for ascertaining PNS were determined pragmatically by analysing descriptive statistics on the frequency of ascertained positive and negative symptoms within the cohort. Patients with missing data in fields related to the inclusion/exclusion criteria were dropped from the analysis.

### Outcomes and other variables

To characterise patients with schizophrenia with PNS, demographic, clinical and treatment information were collected from the NeuroBlu database. Demographic characteristics included sex, age, race/ethnicity, marital status and employment status. ‘Other’ race/ethnicity included Asian, American Indian or Alaska Native, and Native Hawaiian or Other Pacific Islander. Patients with missing race/ethnicity and/or marital status were mapped to ‘Unknown’ for that variable. Clinical characteristics included disease severity measured by the CGI-S score at index, psychiatric comorbidities recorded within 1 year prior to and 7 days post-index, and prescribed medications. CGI-S scores range from 1 to 7 and measure the severity of a patient’s illness relative to the clinician’s experience with patients who have the same diagnosis.^28^ Patients with a missing value for psychiatric comorbidities were assumed not to have the condition. Treatment data included first-line, second-line and third-line antipsychotic therapies, treatment switching and treatment discontinuation within 12 months after the index date. Antipsychotics were categorised as atypical (aripiprazole, asenapine, brexpiprazole, cariprazine, clozapine, iloperidone, lurasidone, olanzapine, paliperidone, quetiapine, risperidone, ziprasidone, amisulpride, sertindole, lumateperone, pimavanserin) or typical (chlorpromazine, fluphenazine, haloperidol, loxapine, molindone, perphenazine, pimozide, thioridazine, thiothixene, trifluoperazine, mesoridazine, flupenthixol, zuclopenthixol, prochlorperazine). The number of days on treatment was calculated as the number of days a patient had an active prescription for an antipsychotic.

The primary outcome of interest was the mean number of psychiatric inpatient hospitalisations during the 12 months after the index date. Inpatient hospitalisation was identified by consecutive days of time spent admitted to an inpatient hospital within one of the mental healthcare centres in the NeuroBlu database. Patients without hospitalisation records despite 12 months of follow-up data were assumed not to have a hospitalisation during the study period. Other outcomes included the mean number of psychiatric outpatient visits during the 12 months after the index date as well as the estimated costs of psychiatric inpatient hospitalisations and outpatient visits per patient. The average costs of psychiatric inpatient hospitalisations and outpatient visits (not specific to schizophrenia) were estimated using the average cost of psychiatry admissions (average spending per visit=$9879) and outpatient psychiatry visits (average spending per visit=$102), respectively, as defined by the Healthcare Cost Institute.^36^

### Statistical analysis

Descriptive statistics were reported at index for the PNS and non-PNS cohorts. Mean and SD as well as median were reported for continuous variables, and frequency and percentage were reported for categorical variables. Statistical tests were t-tests for continuous variables and chi-squared statistics for categorical variables. Treatment patterns were visualised using Sankey diagrams for the first three antipsychotic treatments using prescription length. Sankey diagrams represent a longitudinal visualisation of changes from one variable category to another. The size of each block in the diagram is proportional to the number of patients represented within a given category. In the present study, the first set of blocks represents the first prescribed antipsychotic and subsequent sets represent the second and third prescribed antipsychotic. The streams connecting the first and second and the second and third sets represent changes (if any) between the first and second and the second and third prescribed antipsychotic. The use of Sankey diagrams enables a visual analysis of overall treatment pattern within each analysed cohort.

For the comparisons of HCRU and costs, a modified genetic matching algorithm was used to construct equally sized PNS and non-PNS cohorts^37 38^ to address potential sources of bias and confounding. The algorithm balanced the cohorts on demographic characteristics (age, race/ethnicity), clinical (CGI-S, number of comorbidities) and treatment (drug class) characteristics at index. The Kolmogorov-Smirnov test was used to evaluate the difference between PNS and non-PNS distributions. Chi-squared statistics were computed before and after matching to determine a good balance (ie, characteristics not statistically significantly different between the PNS and non-PNS cohorts). HCRU and costs were compared between the balanced groups using t-tests for continuous variables and chi-squared statistics for categorical variables. All analyses were conducted using Python v3.9.10 (CreateSpace, Scotts Valley, California, USA). Python packages included pandas 1.1.5, numpy 1.22.3, matplotlib 3.5.0 and scipy 1.8.0. Statistical significance was defined as p value <0.05.

## Results

### Patient characteristics

**Figure 1:**
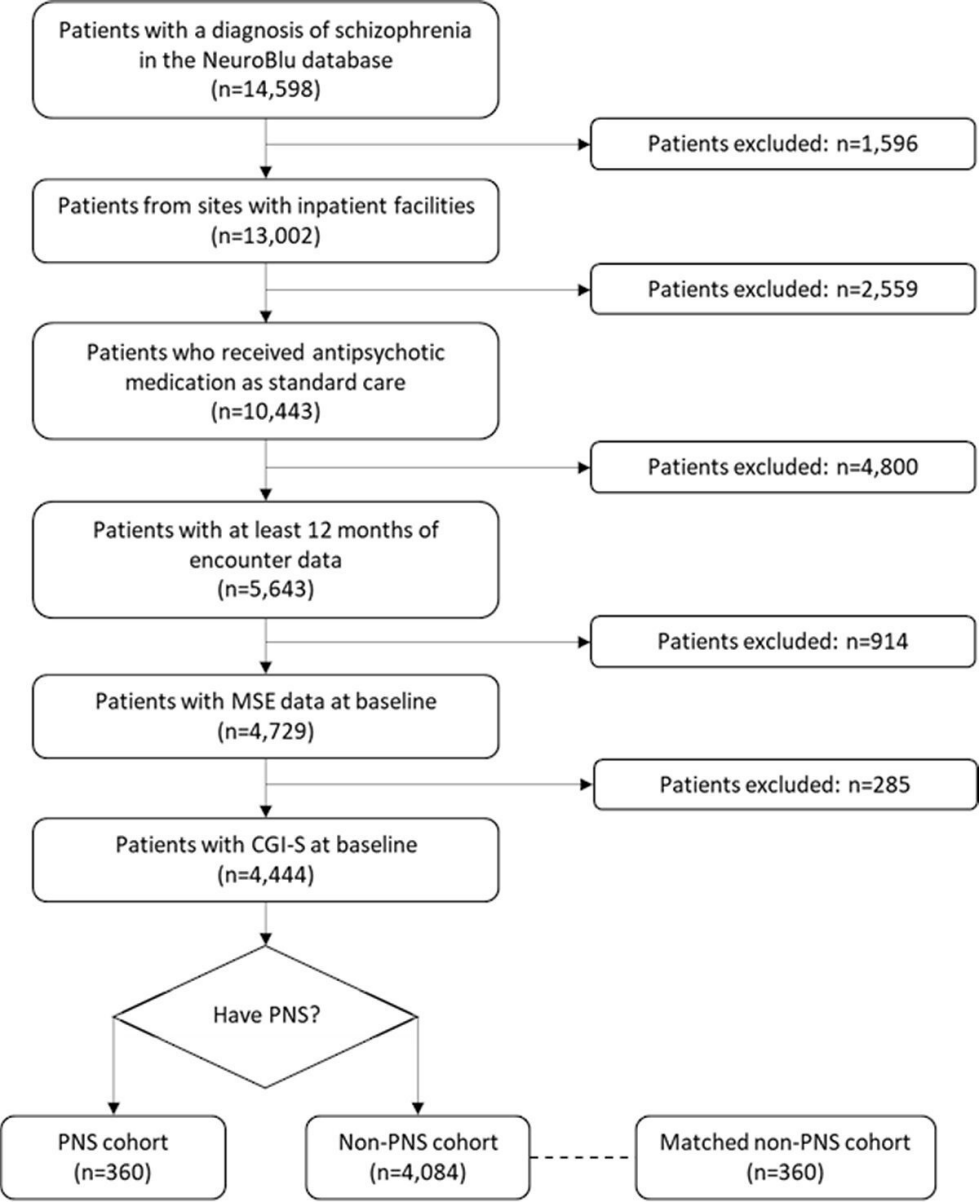
Flow diagram. CGI-S, Clinical Global Impressions – Severity of Illness scale; MSE, mental state examination; n, number of patients; PNS, predominant negative symptoms.

Among the 14 598 patients with a schizophrenia diagnosis in the NeuroBlu database, 4444 patients (30.4%) met the inclusion/exclusion criteria (figure 1). Most patients had an index date between 2008 and 2014 (online supplemental efigure 1).

Of the included patients, 360 (8.1%) were in the PNS cohort and 4084 (91.9%) were in the non-PNS cohort (table 1). Patients with PNS were younger (mean=36.4 vs 39.7 years, p<0.001) and had greater disease severity (mean CGI-S=5.0 vs 4.5, p<0.001) compared with the non-PNS cohort. Patients with PNS also had a greater number of psychiatric comorbidities (mean=1.2 vs 1.0, p<0.001) including greater prevalence of schizoaffective disorders (25.0 vs 18.4%, p=0.003) and major depressive disorder (17.8 vs 9.8%, p<0.001) compared with the non-PNS cohort. Patients with PNS had a greater antipsychotic prescription rate at baseline (no antipsychotic prescription at baseline: 35.8 vs 46.9%, p<0.001). After balancing, the matched non-PNS cohort had 360 patients, and the patient characteristics at baseline were not statistically significantly different (all p≥0.05). Demographic and clinical characteristics after matching are reported in online supplemental etable 2.

**Table 1:** Demographic and clinical characteristics at index

	PNS (n=360)	Non-PNS (n=4084)	P value	P value after match
Age, years, mean (SD) (median)	36.4 (14.4) (35)	39.7 (15.3) (40)	<0.001	0.996
Male, n (%)	242 (67.2)	2556 (62.6)	0.20	--
Race/ethnicity, n (%)			<0.001	0.091
White	124 (34.4)	1695 (41.5)		
Black or African American	156 (43.3)	1332 (32.6)		
Other	31 (8.6)	481 (11.8)		
Unknown	49 (13.6)	576 (14.1)		
Marital status, n (%)			0.06	--
Single	267 (74.2)	2765 (67.7)		
Divorced or separated	26 (7.2)	449 (11.0)		
Married	20 (5.6)	260 (6.4)		
Unknown	47 (13.1)	610 (14.9)		
CGI-S score, mean (SD) (median)	5.0 (1.0) (5)	4.5 (1.2) (5)	<0.001	0.657
Disease severity, n (%)			<0.001	--
Mild (CGI-S=1–3)	26 (7.2)	676 (16.6)		
Moderate (CGI-S=4–5)	222 (61.7)	2653 (65.0)		
Severe (CGI-S=6–7)	112 (31.1)	755 (18.5)		
Number of psychiatric comorbidities, mean (SD) (median)	1.2 (1.3) (1)	1.0 (1.3) (1)	<0.001	0.615
Psychiatric comorbidities, n (%)				
Substance-related disorders	91 (25.3)	933 (22.8)	0.324	--
Schizoaffective disorders	90 (25.0)	753 (18.4)	0.003	--
Bipolar disorder	42 (11.7)	501 (12.3)	0.803	--
Major depressive disorder	64 (17.8)	402 (9.8)	<0.001	--
Personality disorder	26 (7.2)	298 (7.3)	1.000	--
Post-traumatic stress disorder	16 (4.4)	198 (4.8)	0.830	--
Intellectual disabilities	19 (5.3)	190 (4.7)	0.684	--
Generalised anxiety disorder	4 (1.1)	81 (2.0)	0.338	--
Phobic anxiety disorders	6 (1.7)	69 (1.7)	1.000	--
Antipsychotic drug use, n (%)			<0.001	0.309
Atypical antipsychotics	150 (41.7)	1511 (37.0)		
Typical antipsychotics	37 (10.3)	345 (8.4)		
Both atypical and typical antipsychotics	44 (12.2)	314 (7.7)		
None	129 (35.8)	1914 (46.9)		
Number of positive symptoms, n (%)			<0.001	--
0	62 (17.2)	1859 (45.5)		
1	81 (22.5)	858 (21.0)		
2	122 (33.9)	677 (16.6)		
3	95 (26.4)	400 (9.8)		
4	0 (0.0)	192 (4.7)		
5	0 (0.0)	72 (1.8)		
6+	0 (0.0)	26 (0.6)		
Number of negative symptoms, n (%)			<0.001	--
0	0 (0.0)	2056 (50.3)		
1	0 (0.0)	1286 (31.5)		
2	0 (0.0)	668 (16.4)		
3	240 (66.7)	39 (1.0)		
4	84 (23.3)	21 (0.5)		
5	26 (7.2)	9 (0.2)		
6+	10 (2.8)	5 (0.1)		

Notes: Other race/ethnicity included Asian, American Indian or Alaska Native, and Native Hawaiian or Other Pacific Islander.

--variable not included in match--,variable not included in match; CGI-S, Clinical Global Impressions – Severity of Illness scale; n, number of patients; nr, not reportedPNSpredominant negative symptomsSD, standard deviation

### Positive and negative symptoms

**Figure 2:**
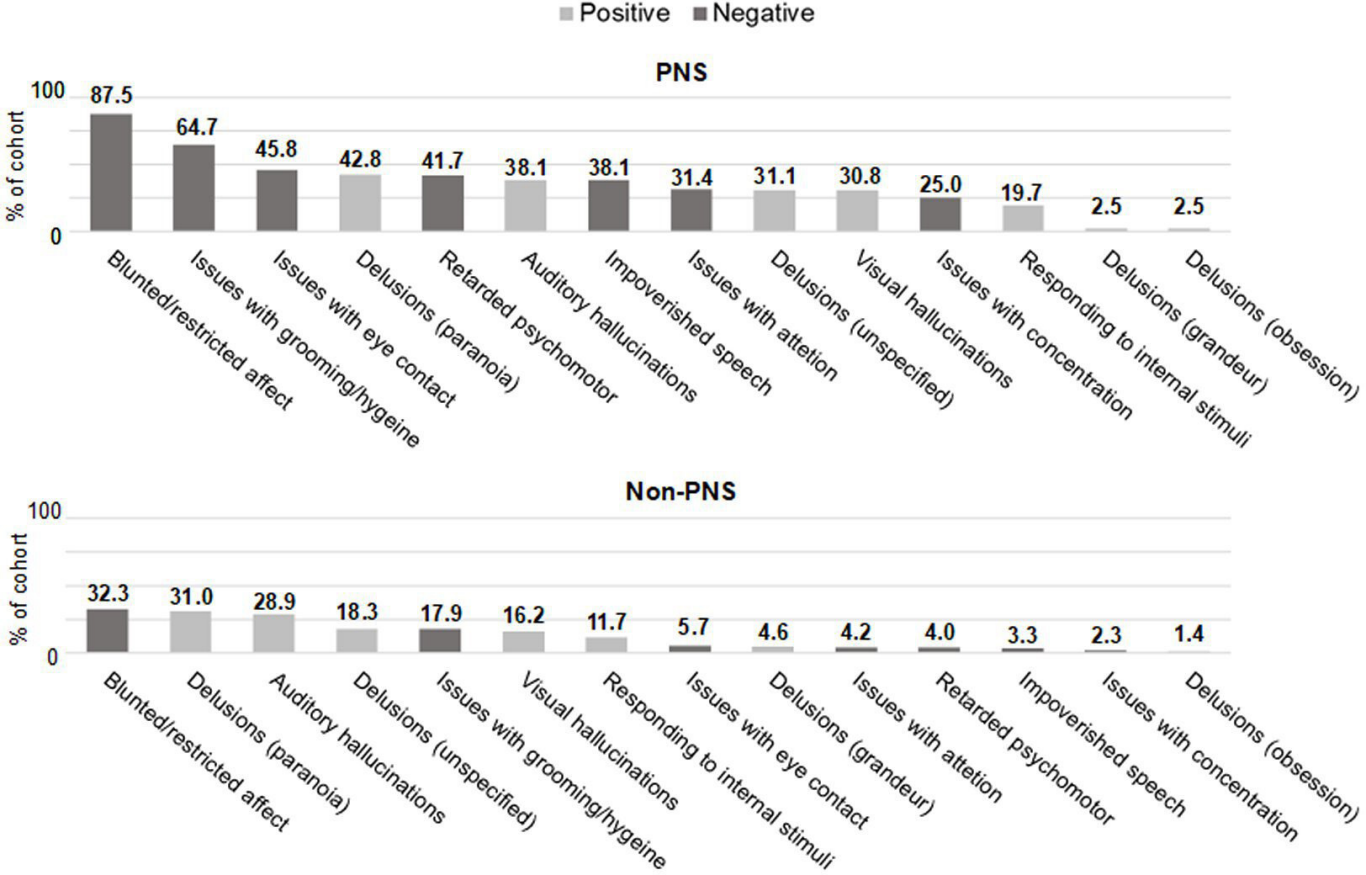
Frequency of positive and negative symptoms. PNS, predominate negative symptoms.

Both the PNS and non-PNS cohorts had a mix of positive and negative symptoms at baseline (figure 2). The most frequent symptom in both cohorts was blunted/restricted affect, a negative symptom. However, patients with PNS had a much greater frequency of blunted/restricted affect compared with the non-PNS cohort (87.5 vs 32.3%). Of the top 5 symptoms in each cohort, 80% (4/5) were negative in the PNS cohort, and 60% (3/5) were positive in the non-PNS cohort.

### Treatment patterns

**Figure 3:**
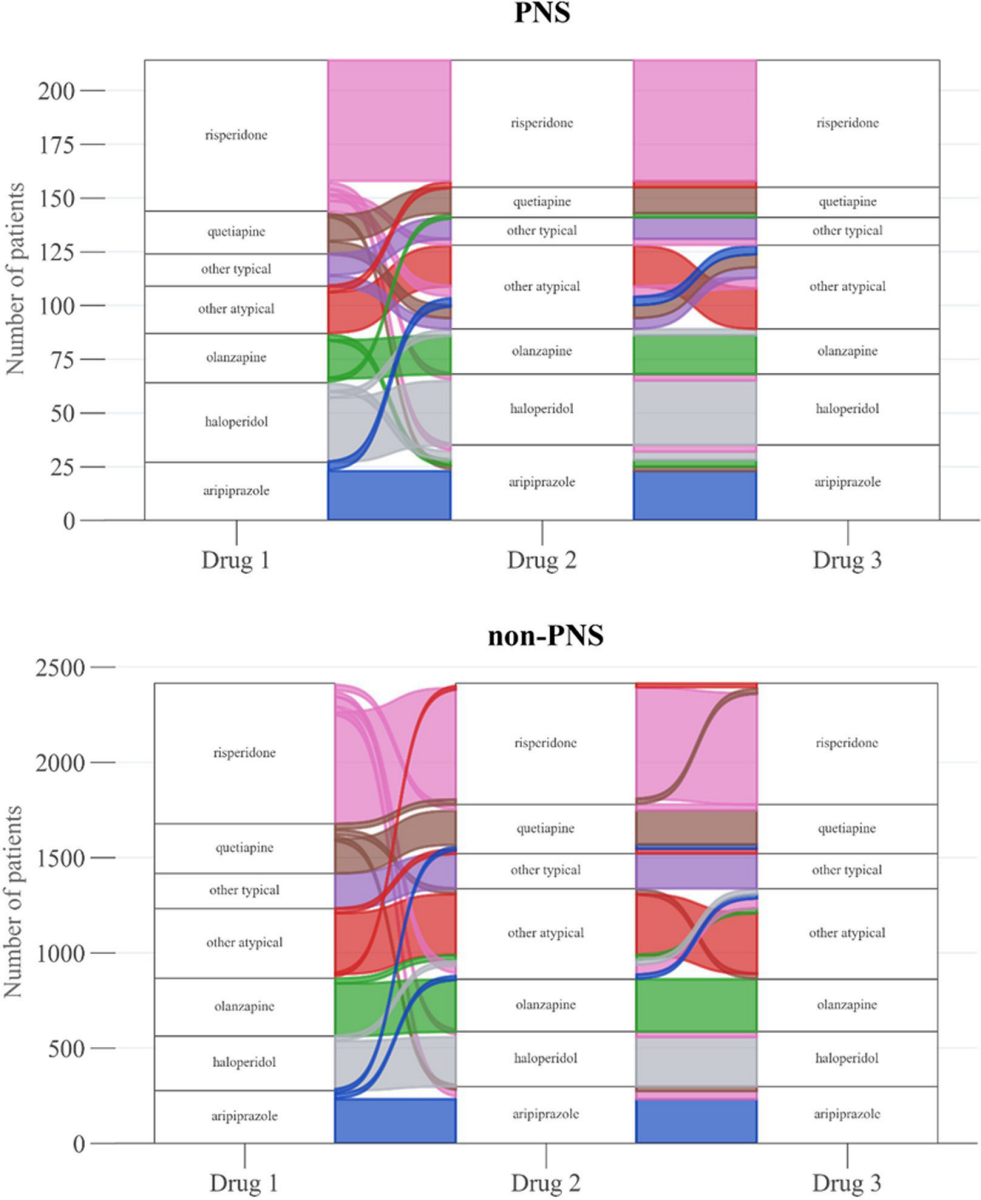
Sankey diagrams. Notes: The Sankey diagrams reflect the first three antipsychotics prescribed within 12 months before or after the index date. Treatment trajectories reflect data from patients with at least three unique antipsychotic prescriptions. Other typical antipsychotics include chlorpromazine, fluphenazine, loxapine, molindone, perphenazine, pimozide, thioridazine, thiothixene, trifluoperazine, mesoridazine, flupenthixol, zuclopenthixol and prochlorperazine. Other atypical antipsychotics include asenapine, brexpiprazole, cariprazine, clozapine, iloperidone, lurasidone, paliperidone, ziprasidone, amisulpride, sertindole, lumateperone and pimavanserin. PNS, predominant negative symptoms.

The top 3 antipsychotics prescribed in the PNS cohort during follow-up were risperidone, aripiprazole and haloperidol (figure 3). The top 3 antipsychotics prescribed in the non-PNS cohort during follow-up were risperidone, olanzapine and other atypical antipsychotics.

By prescription duration during follow-up, the top 3 antipsychotics in the PNS cohort were haloperidol, olanzapine and quetiapine compared with risperidone, quetiapine and olanzapine in the non-PNS cohort (online supplemental etable 3). The mean total duration of treatment with an antipsychotic was lower in the PNS cohort compared with the non-PNS cohort (111.8 vs 140.9 days, p<0.001).

### HCRU and costs

After matching on demographic and clinical characteristics, the mean number of inpatient admissions in the PNS cohort was 1.7 compared with 1.4 in the non-PNS cohort (p=0.04) (table 2). The mean number of outpatient visits was 14.2 in the PNS cohort vs 16.3 in the non-PNS cohort (p=0.03).

**Table 2:** HCRU and costs within the 12 months following index date

	PNS (n=360)	Non-PNS (matched, n=360)	P value
Proportion of patients with psychiatric inpatient hospitalisation, n (%)	274 (76.1)	215 (59.7)	<0.001
Psychiatric inpatient visits, mean (SD) (median)	1.7 (2.0) (1)	1.4 (2.2) (1)	0.04
Number of hospitalisations, n (%)			<0.001
0	86 (23.9)	145 (40.3)	
1	134 (37.2)	109 (30.3)	
2	61 (16.9)	49 (13.6)	
3+	79 (21.9)	57 (15.8)	
Cumulative psychiatric inpatient days, mean (SD) (median)	18.0 (30.0) (8.5)	17.0 (29.4) (4.0)	0.71
Number of inpatient days, n (%)			<0.001
1–10	156 (56.9)	129 (60.0)	
11–20	35 (12.8)	37 (17.2)	
20+	83 (30.3)	49 (22.8)	
Psychiatric inpatient costs, per patient, mean (SD) (median)	$16 893 (19 956) (9879)	$13 732 (21 240) (9879)	0.04
Psychiatric outpatient visits, mean (SD) (median)	14.2 (41.7) (0)	16.3 (29.7) (1)	0.03
Psychiatric outpatient costs, per patient, mean (SD) (median)	$1448 (4249) (0)	$1659 (3025) (102)	nr

HCRUhealthcare resource utilisationn, number of patients; nr, not reported; PNS, predominant negative symptomsSD, standard deviation

A total of 274 patients (76.1%) in the PNS cohort had any psychiatric inpatient hospitalisation compared with 215 (59.7%) patients in the non-PNS cohort (p<0.001). Among patients with any inpatient hospitalisation, 149 patients (38.9%) in the PNS cohort had two or more hospitalisations compared with 106 patients (29.4%) in the non-PNS cohort. The PNS cohort had a greater proportion of patients with 20 or more days hospitalised during follow-up (30.3 vs 22.8%, p<0.001). The estimated costs for psychiatric inpatient stays were $16 893 in the PNS cohort compared with $13 732 in the non-PNS cohort (p=0.04).

## Discussion

Patients with schizophrenia with PNS were, on average, younger and had a greater prevalence of psychiatric comorbidities at baseline compared with patients with schizophrenia without PNS. The PNS cohort had fewer days with an antipsychotic prescription during follow-up. In a matched comparison, the PNS cohort had greater average psychiatric inpatient hospitalisations compared with the non-PNS cohort.

The course of schizophrenia varies by age and sex; the peak incidence for males occurs in their early 20s while incidence for females is less peaked over the life course.^2^ Earlier characterisations of patients with schizophrenia with any negative symptoms (not limited to PNS) have reported a greater prevalence of negative symptoms in males and older patients (>40/45 years old for males/females).^7^ Another study comparing cohorts with prominent negative symptoms, prominent positive symptoms and neither prominent negative nor positive symptoms reported no significant difference between cohorts in age, sex, race or marital status.^21^ Although sex, race and marital status were not significantly different in this study, the PNS cohort had a lower average age compared with the non-PNS cohort. Additional differences between the two cohorts included greater prevalence of comorbid depression in patients with PNS. In clinical practice, depressive symptoms and primary negative symptoms may be difficult to distinguish.^6^ Therefore, the difference in frequency of comorbid depression may reflect the presence of negative symptoms.^39^

This study focused on the pharmacological treatment of schizophrenia; psychological treatment approaches may also be indicated for schizophrenia but data on these were not available for analysis.^40^ Although no individual antipsychotic is recommended for the treatment of schizophrenia with PNS,^16^ the European Psychiatric Association guidance recommends that a switch to atypical antipsychotics should be considered for patients with schizophrenia with negative symptoms who are currently being treated with a typical antipsychotic.^6^ In this study, the top prescribed antipsychotic by treatment duration in patients with PNS was a typical antipsychotic (haloperidol) which has been previously shown to be associated with secondary negative symptoms in a placebo-controlled randomised trial.^41^ The definition of PNS in this study with limited or controlled positive symptoms (ie ≥3 negative symptoms and ≤3 positive symptoms) was selected to limit the potential influence of secondary negative symptoms related to positive symptoms in the PNS cohort. However, given the time window during which data were ascertained during the present study (1999–2020), this finding may be affected by the more widespread use of typical antipsychotics in the earlier part of the dataset when atypical antipsychotics may not have been widely available. The results of the present study also suggest that patients with PNS may have struggled to find a treatment that was effective (ie, more treatment switching, shorter treatment duration). More research is needed to develop an effective treatment for negative symptoms in patients with schizophrenia with PNS whose symptoms may be even harder to treat than negative symptoms in patients who do not have PNS.

This study adds to the estimates of HCRU and inpatient healthcare costs for patients with negative symptoms. A recent systematic literature review reported an association between greater negative symptom burden and increased HCRU and costs.^26^ However, the small number of studies had considerable heterogeneity (ie, differences in patient characteristics, country of study, year of study and included costs), which contributed to a sizeable range in annual direct healthcare costs associated with negative symptoms.^26^ Based on the limited and wide-ranging estimates in the literature, our estimate of psychiatric inpatient costs is likely conservative. Additionally, greater average outpatient visits but fewer inpatient stays among patients without PNS in this study suggest the possibility of substitution of outpatient visits for inpatient stays in the non-PNS cohort.

There were several limitations in this study. As with any EHR database analysis, EHR-derived cohort comparisons are limited to controlling for measured and known confounders that are available in the EHR database. Although we conducted a matched analysis to account for potential demographic and clinical factors in the associations of PNS with HCRU and costs, we did not conduct a separate matched analysis on treatment patterns and it is possible that confounding factors may have influenced the associations of PNS with choice of antipsychotic treatment. Additionally, EHR data are collected for clinical care and repurposed for retrospective analysis meaning that misclassification and incomplete or delayed data entry might be inherent in the data, particularly, in relation to symptom data which may not always be comprehensively documented. As the primary outcome measure for the study was the number of psychiatric hospitalisations during the follow-up period, we included patients who were receiving care from clinical sites with psychiatric hospital facilities. For this reason, it is not possible to generalise findings to patients receiving care from clinical sites without psychiatric hospital facilities and the requirement for clinic sites to have psychiatric hospital facilities may have introduced selection bias into the assembled cohort. Patients may have received care from other healthcare providers not represented in the NeuroBlu database. Furthermore, patients receiving care from the healthcare providers represented in the NeuroBlu database may have received their first episode of care prior to the implementation of EHRs while paper clinical records were still in use. This means that the first recorded diagnosis of schizophrenia may not represent the first episode of schizophrenia and the cohort included in the study is likely to include patients who already had chronic schizophrenia prior to the index date. Furthermore, as formal diagnostic assessment tools are not readily implemented in real-world clinical practice, the application of diagnostic criteria for schizophrenia may have varied between different clinical sites. The structure of the EHR system employed by healthcare providers in real-world clinical practice was predetermined, so we did not choose how to categorise clinical features as part of the MSE. In the NeuroBlu database, the NLP-derived MSE labels used in this study are derived from clinician notes and have not been externally validated. Furthermore, NeuroBlu data does not capture HCRU in mental healthcare centres outside of the NeuroBlu database. The analysis was limited to patients from NeuroBlu mental healthcare centres with inpatient facilities. Nonetheless, it is possible that hospitalisations to other mental healthcare centres were missed. The NeuroBlu database did not include healthcare-related cost information, so we estimated the economic burden using the average cost of psychiatric hospitalisations and outpatient visits. The burden could be greater if the cost of schizophrenia-related hospitalisations and outpatients visits is greater than general psychiatric hospitalisations and outpatient visits.

There is often significant heterogeneity between different clinical centres in real-world clinical practice, which is likely to have impacted the level of attrition related to the inclusion criteria. Changes over time may have also influenced HCRU and treatment patterns. As the dataset comprised real-world data, the sample sizes were not uniform across different year groups, so we were unable to conduct analyses stratifying by time. Another limitation is that the antipsychotic treatment dose, which may be associated with negative symptoms in patients with schizophrenia,^7^ was not captured in the analysis. Finally, the point-in-time measurement of symptoms and disease severity at index may not be representative of the patient’s experience over the follow-up period. The point-in-time measurement of symptoms also excluded the inclusion of motivational or experiential negative symptoms, which are identified over time and are less likely to be represented within the MSE compared with the clinical history. Due to patient confidentiality and data governance restrictions, clinical history data are not available in the NeuroBlu database. Nevertheless, the limitations of analysing real-world data are balanced by the potential to analyse data from a large sample size that is greater than what would be feasible through a prospective study.

In summary, we found that patients with schizophrenia with PNS were younger and had more psychiatric comorbidities compared with patients without PNS. There was some evidence of greater HCRU in patients with schizophrenia with PNS, which could indicate a greater burden and unmet need for the treatment of negative symptoms. New therapies that specifically address negative symptoms, particularly, among individuals with PNS, could improve clinical outcomes and reduce HCRU.

## supplementary material

10.1136/bmjopen-2024-084613 online supplemental file 1

## Data Availability

No data are available.
