# Supplementary material for: A real-world data analysis of electronic health records to investigate the associations of predominant negative symptoms with healthcare resource utilisation, costs and treatment patterns among patients with schizophrenia
Source: BMJ Open. 2024 Jul 31;14(7):e084613. doi: 10.1136/bmjopen-2024-084613 (PMC11293408; doi:10.1136/bmjopen-2024-084613)
Supplement: online supplemental file 1 [file bmjopen-14-7-s001.pdf]

Supplementary Materials: A real-world data analysis of electronic health records to investigate the associations of predominant negative symptoms with healthcare resource utilization, costs and treatment patterns among patients with schizophrenia

Rashmi Patel[1]*, Carole Dembek[2], Yida Won[3], Aditi Kadakia[2], Xueyan Huang[3], Courtney Zeni[2], Andrei Pikalov[4]

[1] Department of Psychiatry, University of Cambridge, Cambridge, UK;

[2] Sunovion Pharmaceuticals Inc., Marlborough, MA, USA;

[3] Holmusk Technologies Inc., New York, NY, USA;

[4] APILAX LLC, Kinnelon, NJ, USA

*** Corresponding author:**

Rashmi Patel

Herchel Smith Building for Brain and Mind Sciences

Department of Psychiatry

University of Cambridge

Robinson Way

Cambridge CB2 0SZ

United Kingdom

Email: rkrp2@cam.ac.uk

eTable 1. NLP-derived MSE features mapped to positive and negative symptoms

**Symptom type**	**MSE feature**
Negative Symptoms	Affect: Blunted/restricted
	Appearance: Issues with grooming/hygiene; eye contact; dress
	Attention & concentration: Declined; issues due to mental health
	Cognition: Issues with concentration; issues with attention
	Language: Non-verbal/mute; minimally verbal
	Psychomotor: Retarded; slowed; declined; catatonic
	Speech: Impoverished; mute; non-verbal
Positive Symptoms	Hallucinations: history of hallucinations; experiencing auditory/visual/olfactory/tactile/unspecified hallucinations
	Delusions: history of delusions or abnormal thoughts; experiencing delusions or abnormal thoughts of paranoia/persecution/grandeur/religious nature/obsessive nature/sexual nature/not specified
	Other: responding to internal stimuli

Abbreviations: MSE, mental state examination; NLP, natural language processing.

eTable 2. Demographic and clinical characteristics at index after match

	PNS (n=360)	Non-PNS (n=360)	P-value
Age, years, mean (SD)	36.4 (14.4) [35]	40.7 (15.4) [42]	<0.001
Male, n (%)	242 (67.2)	228 (63.3)	0.308
Race/ethnicity, n (%)			0.028
White	124 (34.4)	155 (43.1)	
Black or African American	156 (43.3)	122 (33.9)	
Other	31 (8.6)	40 (11.1)	
Unknown	49 (13.6)	43 (11.9)	
Marital status, n (%)			0.615
Single	267 (74.2)	264 (73.3)	
Divorced or separated	26 (7.2)	34 (9.4)	
Married	20 (5.6)	15 (4.2)	
Unknown	47 (13.1)	47 (13.1)	
CGI-S score, mean (SD)	5.0 (1.0)	4.5 (1.1)	<0.001
Disease severity, n (%)			<0.001
Mild (CGI-S=1-3)	26 (7.2)	61 (16.9)	
Moderate (CGI-S=4-5)	222 (61.7)	237 (65.8)	
Severe (CGI-S=6-7)	112 (31.1)	62 (17.2)	
Number of psychiatric comorbidities, mean (SD)	1.2 (1.3)	1.1 (1.2)	0.041
Psychiatric comorbidities, n (%)			
Substance-related disorders	91 (25.3)	84 (23.3)	0.602
Schizoaffective disorders	90 (25.0)	73 (20.3)	0.154
Bipolar disorder	42 (11.7)	54 (15.0)	0.228

Major depressive disorder	64 (17.8)	33 (9.17)	0..001
Personality disorder	26 (7.2)	32 (8.9)	0.494
Post-traumatic stress disorder	16 (4.4)	10 (2.8)	0.318
Intellectual disabilities	19 (5.3)	26 (7.2)	0.356
Generalized anxiety disorder	4 (1.1)	5 (1.4)	1.00
Phobic anxiety disorders	6 (1.7)	5 (1.4)	1.00
Antipsychotic drug use, n (%)			0.006
Atypical antipsychotics	150 (41.7)	139 (38.6)	
Typical antipsychotics	37 (10.3)	17 (4.7)	
Both atypical and typical antipsychotics	44 (12.2)	39 (10.9)	
None	129 (35.8)	165 (45.8)	
Number of positive symptoms, n (%)			<0.001
0	62 (17.2)	157 (43.6)	
1	81 (22.5)	86 (23.9)	
2	122 (33.9)	48 (13.3)	
3	95 (26.4)	41 (11.4)	
4	0 (0.0)	23 (6.4)	
5	0 (0.0)	3 (0.8)	
6+	0 (0.0)	2 (0.6)	
Number of negative symptoms, n (%)			<0.001
0	0 (0.0)	158 (43.9)	
1	0 (0.0)	123 (34.2)	
2	0 (0.0)	66 (18.3)	
3	240 (66.7)	6 (1.7)	
4	84 (23.3)	6 (1.7)	
5	26 (7.2)	1 (0.3)	
6+	10 (2.8)	0 (0.0)	

Abbreviations: --, variable not included in match; CGI-S, Clinical Global Impressions – Severity of Illness scale; n, number of patients; nr, not reported; SD, standard deviation.

Notes: Other race/ethnicity included Asian, American Indian or Alaska Native, and Native Hawaiian or Other Pacific Islander.

eTable 3. Treatment patterns during follow-up

	PNS (n=360)	Non-PNS (n=4,084)	P-value
Days with an antipsychotic prescription, mean (SD) [median]	111.8 (111.6) [72]	140.9 (116.5) [126]	<0.001
Switched from first antipsychotic, n (%)	171 (47.5)	1,805 (44.2)	0.249
Days with an antipsychotic prescription by antipsychotic (limited to top 5), mean			nr
Haloperidol	98.3	107.3	
Olanzapine	87.6	107.3	
Quetiapine	84.7	110.2	
Risperidone	83.6	116.9	
Aripiprazole	75.7	105.7	

Abbreviations: n, number of patients; nr, not reported; PNS, predominant negative symptoms; SD, standard deviation.

eFigure 1. Distribution of PNS and non-PNS cohorts by baseline year

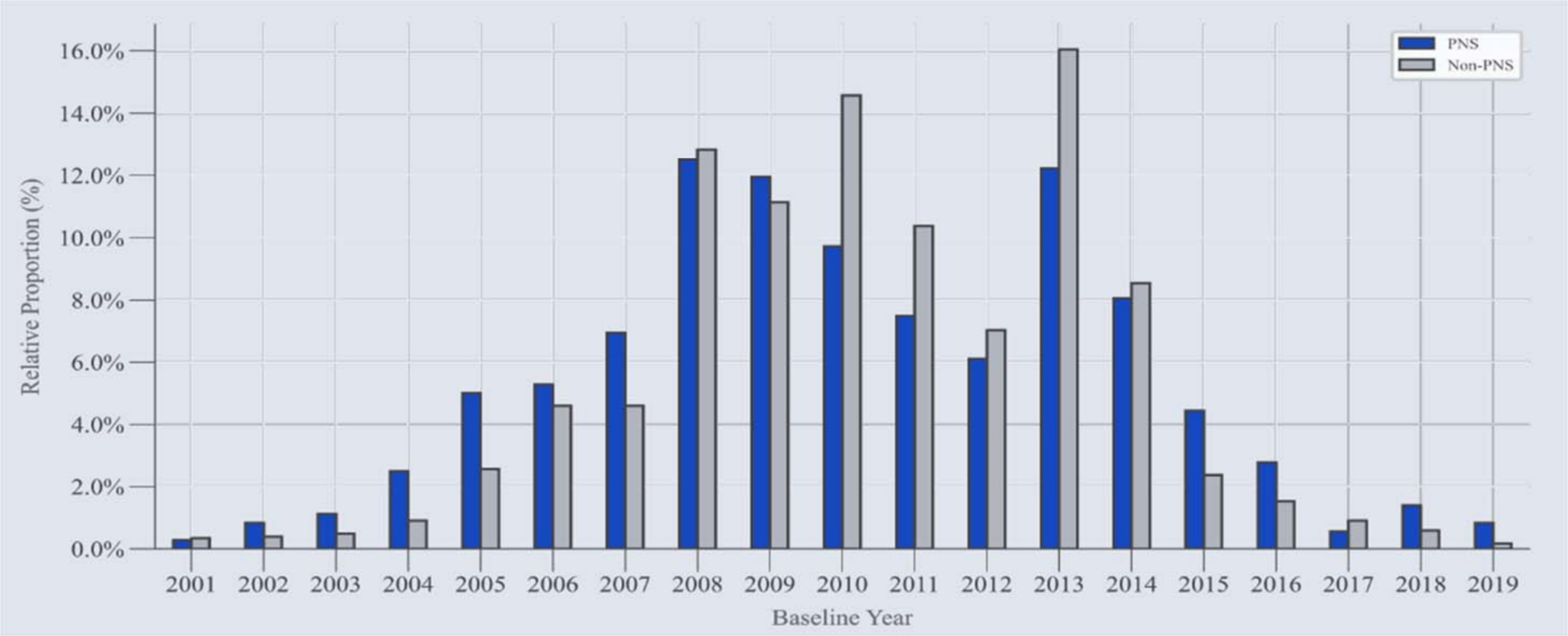

Abbreviations: PNS, predominant negative symptoms.
