# Supplementary material for: A real-world data analysis of electronic health records to investigate the associations of predominant negative symptoms with healthcare resource utilisation, costs and treatment patterns among patients with schizophrenia
Source: BMJ Open. 2024 Jul 31;14(7):e084613. doi: 10.1136/bmjopen-2024-084613 (PMC11293408; doi:10.1136/bmjopen-2024-084613)
Supplement: Supplementary file 2 [file bmjopen-2024-084613.reviewer_comments.pdf]

PEER REVIEW HISTORY

BMJ Open publishes all reviews undertaken for accepted manuscripts. Reviewers are asked to complete a checklist review form (**http://bmjopen.bmj.com/site/about/resources/checklist.pdf**) and are provided with free text boxes to elaborate on their assessment. These free text comments are reproduced below.

ARTICLE DETAILS

**TITLE (PROVISIONAL)**	A real-world data analysis of electronic health records to investigate the associations of predominant negative symptoms with healthcare resource utilization, costs and treatment patterns among patients with schizophrenia
**AUTHORS**	Patel, Rashmi; Dembek, Carole; Won, Yida; Kadakia, Aditi; Huang, Xueyan; Zeni, Courtney; Pikalov, Andrei

VERSION 1 – REVIEW

**REVIEWER**	Jönsson, EG<br>Karolinska Institutet
**REVIEW RETURNED**	26-May-2024

**GENERAL COMMENTS**	The authors have investigated prominent negative symptoms (PNS; defined as ≥3 negative symptoms and ≤3 positive symptoms) among patients with schizophrenia using electronic health care records from 25 private providers of psychiatric healthcare in the USA. In total 4,444 adults with schizophrenia receiving healthcare between 1999 and 2020 were investigated. Of the investigated patients, 8% had prominent negative symptoms and 92% had not. Patients with PNS were younger and had a greater prevalence of concurrent schizoaffective and major depressive disorders. Among the 92% a matched cohort 1:1 was used for further comparisons, showing that PNS patients were more likely to have been hospitalized as inpatients and had greater estimated patient costs.<br><br>This is an interesting well-written report, which seems well performed with regard to methodology. Having read case notes from non-electronic records, it is an interesting opportunity to be able to use electronic health records to get real-life data. There is however a number of caveats with the methodology used, which however have been thoroughly discussed by the authors in a long limitation section in the manuscript. See below some questions.<br><br>1. How does the natural language processing work? Do the algorithms used cover all text data of each patients record? Or are only certain defined areas of the patient record screened for certain questions? From own experience, using other electronic health systems than NeuroBlue, in contrast to diagnosis, dates and patients' identity, which is mandatory to place under certain headlines/boxes, the symptom descriptions may be written not only in certain boxes/headlines aimed for such descriptions, but often at other places in the text, depending on the preferences of each medical doctor. The more stressful situation, the less likely is a good, structured description with all data in correct headlines/boxes in the case note.

	2. What exact words are searched for? Is it those mentioned in eTable 1? Or is eTable 1 more of a description of what is searched for in general and in practice it is a number of specific search strings used for each MSE feature?

**REVIEWER**	Meng, Xiangfei<br>McGill University
**REVIEW RETURNED**	11-Jun-2024

**GENERAL COMMENTS**	The manuscript aimed to test how patients with schizophrenia with negative symptoms are different compared to those without in terms of health care utilization and their antipsychotic treatment patterns. This is an interesting and important topic and it would be beneficial for readers to learn what has been found on negative symptoms beyond costs and healthcare utilization. There are several key issues remaining in the manuscript.<br>The manuscript lacks some key elements, for instance, :1) The introduction of the database analysed is needed in terms of the number of patients, geographic coverage of potential patients, clinical diagnostic criteria, etc. 2) what were the NLP models to be applied? What symptoms were considered in the processing of modelling mental states?<br>Definition of the presence of negative symptoms: What is the justification for choosing >3 symptoms as patients with negative symptoms? The introduction highlighted the issues of inconsistent definitions for patients with negative symptoms. Would patients with more than 3 negative symptoms but more than 3 positive symptoms be treated as patients with negative symptoms? This category of patients was not defined in the methods.<br>It is crucial to learn how variations in service providers (health care costs, institutions/ sites, diagnostic criteria, severity, etc.) were considered when addressing the two research questions.<br>The thing that strikes me the most is the selection process of the study sample. The way the study cohort was selected introduced selection biases, for instance, only those patients being treated at the sites with inpatient services had the data on MSE, CGI-S, and more. How comparable is the remaining cohort compared to the initial and each selection steps? The results are biased given the way of the sample being selected and no such comparison was conducted to explore any potential impact on the results.<br>Another thing that the authors discussed briefly was the potential confounding. As the manuscript did not provide information on whether the matching process was also done for the first objective. We don't know whether the comparison between patients with PNS and those without PNS in terms of their healthcare costs is already considered confounding.

VERSION 1 – AUTHOR RESPONSE

Reviewer: 1
Dr. EG Jönsson, Karolinska Institutet
Comments to the Author:
The authors have investigated prominent negative symptoms (PNS; defined as ≥3 negative symptoms and ≤3 positive symptoms) among patients with schizophrenia using electronic health care records from 25 private providers of psychiatric healthcare in the USA. In total 4,444 adults with schizophrenia receiving healthcare between 1999 and 2020 were investigated. Of the investigated patients, 8% had prominent negative symptoms and 92% had not. Patients with PNS were younger and had a greater prevalence of concurrent schizoaffective and major depressive disorders. Among the 92% a matched

cohort 1:1 was used for further comparisons, showing that PNS patients were more likely to have been hospitalized as inpatients and had greater estimated patient costs.

This is an interesting well-written report, which seems well performed with regard to methodology. Having read case notes from non-electronic records, it is an interesting opportunity to be able to use electronic health records to get real-life data. There is however a number of caveats with the methodology used, which however have been thoroughly discussed by the authors in a long limitation section in the manuscript. See below some questions.

/*Thank you for your supportive comments.*/

1. How does the natural language processing work? Do the algorithms used cover all text data of each patients record? Or are only certain defined areas of the patient record screened for certain questions? From own experience, using other electronic health systems than NeuroBlue, in contrast to diagnosis, dates and patients' identity, which is mandatory to place under certain headlines/boxes, the symptom descriptions may be written not only in certain boxes/headlines aimed for such descriptions, but often at other places in the text, depending on the preferences of each medical doctor. The more stressful situation, the less likely is a good, structured description with all data in correct headlines/boxes in the case note.

/*The natural language processing models were developed from and applied to semi-structured free text data related to the Mental State Examination (MSE). The MSE encompasses a real-time assessment of symptoms and signs related to mental disorders and is typically assessed by a mental health clinician at the initial clinical presentation and thereafter at review appointments. The MindLinc electronic health record system (from which data were obtained for this study) contains a field to record MSE through short free-text descriptions recorded following a clinical encounter. The presence of a dedicated entry field for MSE in the dataset enabled the development of NLP algorithms to identify recorded clinical features that were specific to the presence of positive symptoms and/or negative symptoms. Details of the NLP model development and application to the MindLinc dataset in NeuroBlu are provided in two previous publications:

Patel R, Wee SN, Ramaswamy R, et al. NeuroBlu, an electronic health record (EHR) trusted research environment (TRE) to support mental healthcare analytics with real-world data. BMJ Open 2022;12:e057227. doi:10.1136/bmjopen-2021-057227

Mukherjee SS, Yu J, Won Y, et al. Natural Language Processing-Based Quantication of the Mental State of Psychiatric Patients. Comput Psychiatry 2020;4:76. doi:10.1162/cpsy_a_00030

We have updated the methods section of the manuscript (now labelled as section "3.2 NLP-derived symptom data") to provide further details of NLP model development and application as follows:

"NLP algorithms have been shown to be useful to obtain data on symptoms when semi-structured and/or unstructured text is used to record a patient's mental and behavioral health state.[30] NLP is particularly useful to obtain data that may not be recorded in a standardized way within an EHR dataset. For example, in routine clinical practice clinicians may seldom employ structured rating scales to assess negative symptoms at every clinical assessment but may document the presence of problematic negative symptoms in free text in the MSE section of the EHR. The use of NLP therefore enables the ascertainment of negative symptoms in real-world EHR data where they are documented in free text but where no negative symptom rating scale has been applied. Numerous studies have been published using NLP to ascertain symptoms from EHR data.[8,9,11,30–34]

We applied previously developed natural language processing (NLP) models to identify the presence of seven positive (hallucinations including visual or auditory; delusions including paranoia, grandeur, obsession, or unspecified; responding to internal stimuli) and seven negative symptoms (blunted affect, appearance, attention & concentration, cognition, language, psychomotor, speech) recorded within semi-structured MSE data in the NeuroBlu database using a deep learning approach.[29] In brief, MSE data on 241 clinical features were reorganized into 27 categories representing key features of mental disorders (e.g. "Abnormal or psychotic thoughts", "Mood", "Speech" etc). Free text data recorded in the MSE were tokenized and pre-processed to remove common parts of speech and correct spelling errors. A training dataset was analyzed using a long short-term memory (LSTM) approach with five-fold cross-validation. Models for NLP-derived clinical features were validated against pre-labelled test data. The median AUROC across all clinical features was 0.9 indicating good classification accuracy."

We agree that the accurate ascertainment of symptom data relies on clinicians documenting their presence at the time of clinical assessment and in real-world clinical practice, comprehensive documentation is not guaranteed. We have updated the limitations section of the manuscript to highlight this point:

"Additionally, EHR data are collected for clinical care and repurposed for retrospective analysis meaning that misclassification and incomplete or delayed data entry might be inherent in the data, particularly in relation to symptom data which may not always be comprehensively documented."*/

2. What exact words are searched for? Is it those mentioned in eTable 1? Or is eTable 1 more of a description of what is searched for in general and in practice it is a number of specific search strings used for each MSE feature?

/*Data from 22 clinical features across 10 MSE categories were included in the study. Each clinical feature may include a range of examples identified through NLP models validated against pre-labelled test datasets. Given the scale of the dataset and due to data governance restrictions, it is not possible to provide a list of all documented examples of positive and negative symptoms included in the study and eTable 1 provides a list of the names of the clinical features and categories included in the study. We have updated the methods section 3.2 accordingly:

"Of the 241 clinical features available for analysis, 22 features across 10 categories that were relevant to positive and negative symptoms were ascertained for this study. A summary of the symptom data obtained in the study is included in Supplementary Materials eTable 1."*/

Reviewer: 2
Dr. Xiangfei Meng, McGill University
Comments to the Author:

The manuscript aimed to test how patients with schizophrenia with negative symptoms are different compared to those without in terms of health care utilization and their antipsychotic treatment patterns. This is an interesting and important topic and it would be beneficial for readers to learn what has been found on negative symptoms beyond costs and healthcare utilization. There are several key issues remaining in the manuscript.

/*Thank you for your supportive comments.*/

The manuscript lacks some key elements, for instance, :1) The introduction of the database analysed is needed in terms of the number of patients, geographic coverage of potential patients, clinical

diagnostic criteria, etc. 2) what were the NLP models to be applied? What symptoms were considered in the processing of modelling mental states?

/*In response to your first point, we have previously published a cohort profile of the NeuroBlu dataset in BMJ Open:

Patel R, Wee SN, Ramaswamy R, et al. NeuroBlu, an electronic health record (EHR) trusted research environment (TRE) to support mental healthcare analytics with real-world data. BMJ Open 2022;12:e057227. doi:10.1136/bmjopen-2021-057227

Additionally, we have updated the methods section of the manuscript (3.1 Data source) to provide more details about the population represented within the dataset:

"This retrospective analysis used data from the NeuroBlu platform, a database of de-identified electronic health record (EHR) data from 25 private mental healthcare centers (outpatient, inpatient, telemedicine, and/or residential care facilities) in the United States that employed the MindLinc EHR system.[27] The database covers approximately 560,000 patients treated by over 11,000 clinicians across 12 US states spanning a 21 year period (between 1999 and 2020). Around 14,500 patients in the database have received a diagnosis of schizophrenia. Further details of the mental healthcare centers included in the study are provided in a previously published cohort profile.[27]"

In response to your second point, we have provided additional information in response to Dr. EG Jönsson and updated the methods section of the manuscript (3.2 NLP-derived symptom data) as follows:

"NLP algorithms have been shown to be useful to obtain data on symptoms when semi-structured and/or unstructured text is used to record a patient's mental and behavioral health state.[30] NLP is particularly useful to obtain data that may not be recorded in a standardized way within an EHR dataset. For example, in routine clinical practice clinicians may seldom employ structured rating scales to assess negative symptoms at every clinical assessment but may document the presence of problematic negative symptoms in free text in the MSE section of the EHR. The use of NLP therefore enables the ascertainment of negative symptoms in real-world EHR data where they are documented in free text but where no negative symptom rating scale has been applied. Numerous studies have been published using NLP to ascertain symptoms from EHR data.[8,9,11,30–34]

We applied previously developed natural language processing (NLP) models to identify the presence of seven positive (hallucinations including visual or auditory; delusions including paranoia, grandeur, obsession, or unspecified; responding to internal stimuli) and seven negative symptoms (blunted affect, appearance, attention & concentration, cognition, language, psychomotor, speech) recorded within semi-structured MSE data in the NeuroBlu database using a deep learning approach.[29] In brief, MSE data on 241 clinical features were reorganized into 27 categories representing key features of mental disorders (e.g. "Abnormal or psychotic thoughts", "Mood", "Speech" etc). Free text data recorded in the MSE were tokenized and pre-processed to remove common parts of speech and correct spelling errors. A training dataset was analyzed using a long short-term memory (LSTM) approach with five-fold cross-validation. Models for NLP-derived clinical features were validated against pre-labelled test data. The median AUROC across all clinical features was 0.9 indicating good classification accuracy.

Of the 241 clinical features available for analysis, 22 features across 10 categories that were relevant to positive and negative symptoms were ascertained for this study. A summary of the symptom data obtained in the study is included in Supplementary Materials eTable 1."*/

Definition of the presence of negative symptoms: What is the justification for choosing >3 symptoms as patients with negative symptoms? The introduction highlighted the issues of inconsistent definitions for patients with negative symptoms. Would patients with more than 3 negative symptoms but more than 3 positive symptoms be treated as patients with negative symptoms? This category of patients was not defined in the methods.

/*The study specifically addresses the question of the degree to which predominant negative symptoms (i.e. a clinical presentation that is characterised by a greater burden of negative symptoms than positive symptoms) is associated with healthcare resource utilization and treatment patterns. For this reason, we did not consider patients with more than 3 negative symptoms and more than 3 positive symptoms to have predominant negative symptoms. We chose the threshold of ≥3 recorded negative symptoms and ≤3 recorded positive symptoms pragmatically to maximise the sample size of patients with evidence of negative symptom burden greater than that of positive symptom burden as outlined in the methods section (3.5 Study participants):

"Patients were then categorized as having schizophrenia with PNS if they had ≥3 recorded negative symptoms and ≤3 recorded positive symptoms at index (PNS cohort) or schizophrenia without PNS if they had <3 recorded negative symptoms or >3 recorded positive symptoms at index (non-PNS cohort). The thresholds for ascertaining PNS were determined pragmatically by analyzing descriptive statistics on the frequency of ascertained positive and negative symptoms within the cohort."*/

It is crucial to learn how variations in service providers (health care costs, institutions/sites, diagnostic criteria, severity, etc.) were considered when addressing the two research questions.

/*Healthcare costs were estimated based on unit costs for psychiatric inpatient hospitalizations and outpatient visits using the 2018 Health Care Cost and Utilization Report (https://healthcostinstitute.org/hcci-originals-dropdown/all-hcci-reports/2018-health-care-cost-and-utilization-report). A reference is provided in the methods section of the manuscript (3.6 Outcomes and other variables):

"The average costs of psychiatric inpatient hospitalizations and outpatient visits (not specific to schizophrenia) were estimated using the average cost of psychiatry admissions (average spending per visit=$9,879) and outpatient psychiatry visits (average spending per visit=$102), respectively, as defined by the Health Care Cost Institute.[37]"

We acknowledge that using average costs may over or underestimate the true costs which may vary between healthcare providers. We have commented on this in the limitations section of the manuscript:

"The NeuroBlu database did not include healthcare-related cost information, so we estimated the economic burden using the average cost of psychiatric hospitalizations and outpatient visits. The burden could be greater if the cost of schizophrenia-related hospitalizations and outpatients visits is greater than general psychiatric hospitalizations and outpatient visits."

We also acknowledge that diagnostic criteria may vary between different clinical sites as formal diagnostic screening tools are not typically implemented in real-world clinical practice. We have added the following comment in the limitations section to acknowledge this point:

"Furthermore, as formal diagnostic assessment tools are not readily implemented in real-world clinical practice, the application of diagnostic criteria for schizophrenia may have varied between different clinical sites."

To account for potential variation in disease severity, we included baseline CGI-S in a modified Genetic Matching algorithm. We have commented on this in the methods section (3.7 Statistical analysis):

“For the comparisons of HCRU and costs, a modified Genetic Matching algorithm was used to construct equally sized PNS and non-PNS cohorts[38,39] to address potential sources of bias and confounding. The algorithm balanced the cohorts on demographic characteristics (age, race/ethnicity), clinical (CGI-S, number of comorbidities), and treatment (drug class) characteristics at index.”*/

The thing that strikes me the most is the selection process of the study sample. The way the study cohort was selected introduced selection biases, for instance, only those patients being treated at the sites with inpatient services had the data on MSE, CGI-S, and more. How comparable is the remaining cohort compared to the initial and each selection steps? The results are biased given the way of the sample being selected and no such comparison was conducted to explore any potential impact on the results.

/*We agree that the results of the study may be affected by bias, as is the case for all observational analyses of real-world data.

We had to restrict inclusion to participants receiving care from sites with inpatient services as the primary outcome measure for the study was the number of psychiatric inpatient hospitalizations. It would not have been meaningful to include participants in clinical sites which did not have the facility to provide an inpatient psychiatric service. As MSE and CGI-S were key variables of interest in the study, we could only include patients who had these data recorded. In real-world clinical practice, clinical data may not always be recorded and missing data may contribute to bias when not missing at random. Nevertheless, it would not have been feasible to conduct a prospective study ascertaining the presence of positive and negative symptoms in over 4,400 patients. The advantage of a large sample size is balanced by the limitations of potential for bias and difficulties with missing data that are inherent in real-world data. We have discussed the limitations of missing data in the discussion section and added additional comments related to the potential for bias due to the selection criteria for the study.

“There were several limitations in this study. As with any EHR database analysis, EHR-derived cohort comparisons are limited to controlling for measured and known confounders that are available in the EHR database. Additionally, EHR data are collected for clinical care and repurposed for retrospective analysis meaning that misclassification and incomplete or delayed data entry might be inherent in the data, particularly in relation to symptom data which may not always be comprehensively documented. As the primary outcome measure for the study was the number of psychiatric hospitalizations during the follow-up period, we included patients who were receiving care from clinical sites with psychiatric hospital facilities. For this reason, it is not possible to generalize findings to patients receiving care from clinical sites without psychiatric hospital facilities and the requirement for clinic sites to have psychiatric hospital facilities may have introduced selection bias into the assembled cohort. Patients may have received care from other healthcare providers not represented in the NeuroBlu database. Furthermore, patients receiving care from the healthcare providers represented in the NeuroBlu database may have received their first episode of care prior to the implementation of EHRs while paper clinical records were still in use. This means that the first recorded diagnosis of schizophrenia may not represent the first episode of schizophrenia and the cohort included in the study is likely to include patients who already had chronic schizophrenia prior to the index date. Furthermore, as formal diagnostic assessment tools are not readily implemented in real-world clinical practice, the application of diagnostic criteria for schizophrenia may have varied between different clinical sites. The structure of the EHR system employed by healthcare providers in real-world clinical practice was

predetermined, so we did not choose how to categorize clinical features as part of the MSE. In the NeuroBlu database, the NLP-derived MSE labels used in this study are derived from clinician notes and have not been externally validated. Furthermore, NeuroBlu data does not capture HCRU in mental healthcare centers outside of the NeuroBlu database. The analysis was limited to patients from NeuroBlu mental healthcare centers with inpatient facilities. Nonetheless, it is possible that hospitalizations to other mental healthcare centers were missed. The NeuroBlu database did not include healthcare-related cost information, so we estimated the economic burden using the average cost of psychiatric hospitalizations and outpatient visits. The burden could be greater if the cost of schizophrenia-related hospitalizations and outpatients visits is greater than general psychiatric hospitalizations and outpatient visits.

There is often significant heterogeneity between different clinical centers in real-world clinical practice, which is likely to have impacted the level of attrition related to the inclusion criteria. Changes over time may have also influenced HCRU and treatment patterns. As the dataset comprised real-world data, the sample sizes were not uniform across different year groups, so we were unable to conduct analyses stratifying by time. Another limitation is that the antipsychotic treatment dose, which may be associated with negative symptoms in patients with schizophrenia,[7] was not captured in the analysis. Finally, the point-in-time measurement of symptoms and disease severity at index may not be representative of the patient's experience over the follow-up period. The point-in-time measurement of symptoms also excluded the inclusion of motivational or experiential negative symptoms, which are identified over time and are less likely to be represented within the MSE compared to the clinical history. Due to patient confidentiality and data governance restrictions, clinical history data are not available in the NeuroBlu database. Nevertheless, the limitations of analyzing real-world data are balanced by the potential to analyze data from a large sample size that is greater than what would be feasible through a prospective study."*/

Another thing that the authors discussed briefly was the potential confounding. As the manuscript did not provide information on whether the matching process was also done for the first objective. We don't know whether the comparison between patients with PNS and those without PNS in terms of their healthcare costs is already considered confounding.

/*We agree that confounding factors may have influenced the associations of PNS with clinical outcomes. For this reason, we conducted a matched analysis of HCRU and costs to account for the potential confounding factors of age, race/ethnicity, clinical severity (CGI-S and number of comorbidities) and antipsychotic drug class ascertained at index. However, confounding factors may have also influenced the associations of PNS with antipsychotic treatment patterns. We have commented on this in the limitations section:

"Although we conducted a matched analysis to account for potential demographic and clinical factors in the associations of PNS with HCRU and costs, we did not conduct a separate matched analysis on treatment patterns and it is possible that confounding factors may have influenced the associations of PNS with choice of antipsychotic treatment."

VERSION 2 – REVIEW

**REVIEWER**	Jönsson, EG<br>Karolinska Institutet
**REVIEW RETURNED**	05-Jul-2024

**GENERAL COMMENTS**	The authors have now re-revised their manuscript. They have answered satisfactorily to my questions. I have no further comments.
